# Safety and pharmacokinetics of bimagrumab in healthy older and obese adults with body composition changes in the older cohort

**DOI:** 10.1002/jcsm.12639

**Published:** 2020-12-02

**Authors:** Daniel Rooks, Olivier Petricoul, Jens Praestgaard, Michael Bartlett, Didier Laurent, Ronenn Roubenoff

**Affiliations:** ^1^ Translational Medicine Novartis Institutes for BioMedical Research Cambridge MA USA; ^2^ Translational Medicine Novartis Institutes for BioMedical Research Basel Switzerland; ^3^ Novartis Pharmaceuticals Corporation East Hanover NJ USA

**Keywords:** Activin type II receptor, Bimagrumab, Lean body mass, Thigh muscle volume

## Abstract

**Background:**

Bimagrumab prevents activity of myostatin and other negative regulators of skeletal muscle mass. This randomized double‐blind, placebo‐controlled study investigated safety, pharmacokinetics (PK), and pharmacodynamics of bimagrumab in healthy older and obese adults.

**Methods:**

A cohort of older adults (aged 70–85 years) received single intravenous infusions of bimagrumab 30 mg/kg (*n* = 6) or 3 mg/kg (*n* = 6) or placebo (*n* = 4) and was followed for 20 weeks. A second cohort of obese participants [body mass index (BMI) 30–45 kg/m^2^, aged 18–65 years] received a single intravenous infusion of bimagrumab 30 mg/kg (*n* = 6) or placebo (*n* = 2) and was followed for 12 weeks. Outcomes included the safety, tolerability, and PK of bimagrumab, in both cohorts. Measures of pharmacodynamics were performed in the older adult cohort to evaluate the effects of bimagrumab on thigh muscle volume (TMV), total lean body mass (LBM), total fat body mass, and muscle strength.

**Results:**

All 24 randomized participants completed the study. The older adults had a mean (±SD) age of 74.5 ± 3.4 years and BMI of 26.5 ± 3.5 kg/m^2^. The obese participants had a mean (±SD) age of 40.4 ± 11.8 years, weight of 98.0 ± 11.3 kg, and BMI of 34.3 ± 3.9 kg/m^2^. Adverse events in both cohorts were mostly mild. In older adults, most commonly reported adverse events were upper respiratory tract infection, rash, and diarrhoea (each 3/16, 19%). Obese participants reported muscle spasms and rash (both 5/8, 63%) most often. Non‐linearity was observed in the PK concentration profiles of both cohorts due to target‐mediated drug disposition. Bimagrumab 3 and 30 mg/kg increased mean (±SD) TMV (Week 4: 5.3 ± 1.8% and 6.1 ± 2.2%, vs. placebo: 0.5 ± 2.1%, both *P* ≤ 0.02) and LBM (Week 4: 6.0 ± 3.2%, *P* = 0.03 and 2.4 ± 2.2%, vs. placebo: 0.1 ± 2.4%), which were maintained longer with higher dose level, while total fat body mass (Week 4: −2.7 ± 2.9% and −1.6 ± 3.0%, vs. placebo: −2.3 ± 3.2%) decreased from baseline in older adults, with no change in muscle strength.

**Conclusions:**

Bimagrumab was safe and well tolerated and demonstrated similar PK in older and obese adults. A single dose of bimagrumab rapidly increased TMV and LBM and decreased body adiposity in older adults. Muscle hypertrophy and fat loss were sustained with extended drug exposure.

## Introduction

Several members of the transforming growth factor beta superfamily, such as myostatin, activin A, and others, negatively regulate skeletal muscle mass in animals and humans.^1^, ^2^, ^3^ The activin type II receptor (ActRII), acting through Smad 2/3, is the major pathway regulating skeletal muscle size. Modulating muscle growth by perturbing the signalling pathway with a decoy receptor, an anti‐ligand approach, mostly against myostatin, and an ActRII antagonist has been explored over the past 10 years.^4^, ^5^, ^6^

Bimagrumab is a human monoclonal antibody that blocks the ActRIIs, preventing the activity of myostatin and other negative skeletal muscle regulators. In preclinical studies, single and multiple doses of bimagrumab resulted in consistent and significant increases in muscle weight and demonstrated non‐linear pharmacokinetics (PK).^7^ Results from molecular and *in vivo* studies have demonstrated that the dual blockade of ActRIIA and ActRIIB by bimagrumab results in greater muscle hypertrophy than blocking one subunit or ligand (e.g. myostatin).^8^

A single intravenous (i.v.) dose of bimagrumab 3–30 mg/kg in patients with sporadic inclusion body myositis, sarcopenia, disuse atrophy, and chronic obstructive pulmonary disease showed a good safety profile with predictable PK that rapidly increased skeletal muscle mass.^6^, ^9^, ^10^, ^11^ This paper describes the first study to examine the safety, tolerability, and PK in older participants and to compare the pharmacodynamics (PD) of two dose levels of bimagrumab providing different exposure times, by the magnitude and durability of muscle hypertrophy. In addition, data from this study in obese adults address the question of whether the PK of bimagrumab is affected by the quantity of adipose tissue and looks at comparative safety data from drugs with other mechanisms of action on the myostatin–ActRII pathway.

## Methods

### Study design

**Figure 1:**
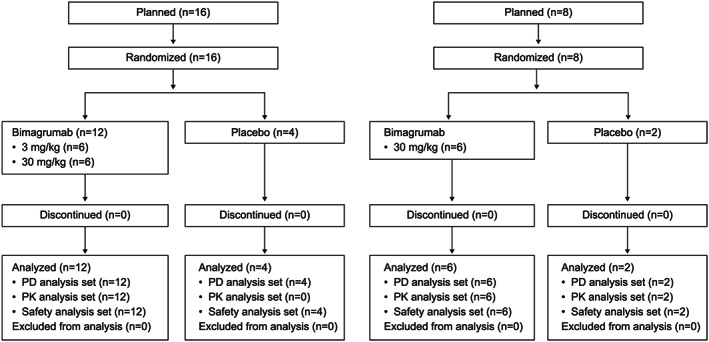
Study participant flow diagram. PD, pharmacodynamics; PK, pharmacokinetics.

This was a Phase I, randomized double‐blind, placebo‐controlled study of bimagrumab in 24 healthy older adult and obese volunteers conducted from February through September 2012 at two centres in the USA. Participants were randomized using a traditional 6:2 design resulting in 18 receiving bimagrumab (*n* = 12, 30 mg/kg and *n* = 6, 3 mg/kg) and six receiving placebo. No restriction on the number of men or women were made because earlier data suggested no differences in safety, PK, or PD between the two sexes. Study drug was administered as a single i.v. infusion of bimagrumab 3 or 30 mg/kg or placebo given over 2 hours (*Figure* 1). The study comprised screening (up to 4 weeks), pre‐dose baseline assessments, a single dose of study drug, and a safety follow‐up period (12 weeks in obese and 20 weeks in older adults). A randomization list was produced by Novartis Drug Supply Management using a validated automated system that randomly assigned participants to one of the treatment arms. All participants, investigators, and sponsor representatives associated with the study were masked to treatment allocation.

The institutional review board at each study site approved the study protocol and amendments. The study was designed, implemented, and reported in accordance with the ICH guidelines for Good Clinical Practice and the principles in the Declaration of Helsinki. All participants provided written informed consent before study initiation.

### Participants

Older adults included healthy men and women aged 70–85 years with a bodyweight of ≥35 kg and body mass index (BMI) of 18–32 kg/m^2^. Obese participants were men and women aged 18–65 years in stable health with a BMI of 30–45 kg/m^2^ and a bodyweight <120 kg. Participants in both cohorts were required to have a stable diet, physical activity or exercise pattern, and bodyweight (±5 kg) within 3 months of screening, and a complete blood count within normal limits. Women were required to be of non‐childbearing potential due to menopause or surgical sterility. Exclusion criteria were presence of cardiac disease or abnormality, history of hypersensitivity to monoclonal antibodies, protein–calorie malabsorption, uncontrolled chronic conditions (i.e. hypertension and type 2 diabetes), diseases known to cause cachexia, and medications or unregulated remedies that could affect skeletal muscle metabolism or turnover. Pregnant or nursing women were also excluded from the study.

### Assessments

The main outcomes focused on the safety, tolerability, and PK of single i.v. infusions of bimagrumab in older and obese adults. Safety was assessed by physical exam, electrocardiogram, changes in laboratory values, and the incidence of adverse events throughout the study. Immunogenicity was assessed by the detection of anti‐drug antibodies. PK parameters [area under the plasma concentration–time curve from time 0 to the time of the last measurable concentration (AUC_last_), maximum plasma concentration (C_max_), and time to reach C_max_ (T_max_)] were derived by non‐compartmental method using WinNonlin Pro Version 5.2. Secondary endpoints included the effect of a single i.v. infusion of bimagrumab on thigh muscle volume (TMV; assessed by magnetic resonance imaging),^12^ lean body mass (LBM) and fat body mass (FBM; assessed by dual‐energy X‐ray absorptiometry),^12^ and muscle strength (assessed by one‐repetition maximum leg press)^13^ in older adults.

### Statistical analysis

Demographics, safety, tolerability, PK, and PD were summarized using descriptive statistics. The effect of treatment with bimagrumab on TMV and LBM was assessed by analysis of covariance on log‐transformed data using the treatment and the log baseline value as covariates. While the study was not powered for showing a treatment effect on the PD endpoint, as the key objectives were safety, tolerability, and PK, we still report *P*‐values for a comparison between treatment and control. These *P*‐values should be viewed in an exploratory fashion as measuring the uncertainty in the estimated treatment effect. No correction for multiplicity of testing was performed. All participants who received one dose of study drug were evaluated for safety and tolerability, and participants treated with bimagrumab who had ≥1 measurable PK concentration were included in the PK analysis set.

## Results

### Participant disposition and baseline characteristics

In total, 16 older adults were randomized and completed the study. Twelve participants received a single i.v. dose of bimagrumab (2 men and 10 women) and four received placebo (three men and one women) (*Figure* 1). The older adult cohort had a mean (±SD) age of 74.5 ± 3.4 years and was predominantly Caucasian (94%) with a mean BMI of 26.5 ± 3.5 kg/m^2^ (*Table* 1). Randomization resulted in baseline characteristic differences between treatment groups. However, none of the differences were considered to influence the primary or secondary outcomes (safety, PK, or PD) assessed in the study.

**Table 1:** Demographics and baseline characteristics of the participants

Characteristics		Older adults			Obese adults	
		Bimagrumab 3 mg/kg (*n* = 6)	Bimagrumab 30 mg/kg (*n* = 6)	Placebo (*n* = 4)	Bimagrumab 30 mg/kg (*n* = 6)	Placebo (*n* = 2)
Age (years)	Mean ± SD	74.5 ± 4.9	73 ± 2.1	76.8 ± 1.0	40.2 ± 12.1	41 ± 15.6
	Range	70–83	71–77	76–78	31–58	30–52
Gender, *n* (%)	Men	0	2 (33.3)	3 (75.0)	5 (83.3)	0
	Women	6 (100.0)	4 (66.7)	1 (25.0)	1 (16.7)	2 (100.0)
Race, *n* (%)	Caucasian	6 (100.0)	5 (83.3)	4 (100.0)	5 (83.3)	2 (100.0)
	Black	0	1 (16.7)	0	1 (16.7)	0 (0.0)
Ethnicity, *n* (%)	Hispanic/Latino	6 (100.0)	6 (100.0)	1 (25.0)	1 (16.7)	1 (50.0)
	Other	0	0	3 (75.0)	5 (83.3)	1 (50.0)
Height (cm)	Mean ± SD	154.1 ± 7.2	161.9 ± 8.2	166.6 ± 10.9	172.3 ± 13.4	161 ± 2.8
	Range	146–165.1	154.9–177	160–182.8	148–189	159–163
Weight (kg)	Mean ± SD	63.7 ± 11.0	75.7 ± 15.4	64.1 ± 4.2	97.8 ± 12.7	98.7 ± 9.4
	Range	51.9–79.5	56.8–101	60.4–69.1	78.8–115.7	92–105.3
BMI (kg/m^2^)	Mean ± SD	26.7 ± 2.6	28.6 ± 3.2	23.3 ± 2.8	33 ± 3.6	38 ± 2.3
	Range	22.2–29.2	23.7–32.2	19.7–26.7	30.1–39.1	36.4–39.6
TMV (cm^3^)	Mean ± SD	2406.5 ± 467.7	2953.2 ± 795.2	3225.2 ± 497.0	NA	NA
Total LBM (kg)	Mean ± SD	29.5 ± 4.5	36.3 ± 7.6	38 ± 3.1	NA	NA
Total FBM (kg)	Mean ± SD	27.4 ± 6.6	31.6 ± 9.1	17.5 ± 3.3	NA	NA
Bilateral leg press (kg)	Mean ± SD	61.6 ± 13.4	59 ± 13.2	48.8 ± 14.4	NA	NA

BMI, body mass index; FBM, fat body mass; LBM, lean body mass; NA, not applicable; SD, standard deviation; TMV, thigh muscle volume.

In the second cohort, eight obese adults were randomized and completed the study. Six participants received a single i.v. dose of bimagrumab (five men and one women) and two received placebo (both women) (*Figure* 1). Obese participants had a mean (±SD) age of 40.4 ± 11.8 years, were predominantly Caucasian (88%), men (63%), and weighed an average of 98.0 ± 11.3 kg, with a mean BMI of 34.3 ± 3.9 kg/m^2^. Baseline characteristics are summarized in *Table* 1.

### Safety

Overall, bimagrumab was safe and well tolerated in this sample of healthy middle‐aged and older adults. There were no adverse events leading to discontinuations from the study and no deaths. The only serious adverse event was an acetabulum fracture that occurred in a participant who received the placebo. Adverse events were mild or moderate in severity (Grade 1; CTCAE),^14^ transient, did not require treatment, and resolved spontaneously within the follow‐up period.

In the older adult cohort, 10/16 (63%) participants experienced ≥1 adverse event (*Table* 2). The most commonly reported adverse events were upper respiratory tract infection, rash, pruritus, papule, and diarrhoea (each 3/16, 19%) followed by gastritis, erythema, and bronchitis (each 2/16, 13%). Muscle‐related symptoms (muscle spasms and muscle pain/myalgia) were reported in 25% (3/12) of participants in the bimagrumab group and none in the placebo group. Overall, adverse events suspected to be related to the study drug were reported in 5/16 (31%) participants, primarily papule and erythema (both 2/16, 13%).

**Table 2:** Adverse events in the safety population

	Older adults		
Summary of AEs	Bimagrumab 3 mg/kg (*n* = 6)	Bimagrumab 30 mg/kg (*n* = 6)	Placebo (*n* = 4)
Patients with any AE	4 (67)	4 (67)	2 (50)
Rash	0	2 (33)	1 (25)
Muscle spasms	1 (17)	0	0
Acetabulum fracture	0	0	1 (25)
Anaemia	1 (17)	0	0
Arthralgia	0	1 (17)	0
Bronchitis	1 (17)	1 (17)	0
Contusion	0	0	1 (25)
Cough	0	1 (17)	0
Dermatitis allergic	1 (17)	0	0
Diarrhoea	1 (17)	2 (33)	0
Dizziness	0	0	1 (25)
Erythema	1 (17)	1 (17)	0
Excoriation	0	0	1 (25)
Gastritis	2 (33)	0	0
Gastroenteritis	0	1 (17)	0
Gastroenteritis viral	1 (17)	0	0
Muscle strain	0	1 (17)	0
Musculoskeletal pain	0	1 (17)	0
Myalgia	0	1 (17)	0
Pain	0	0	1 (25)
Pain of skin	1 (17)	0	0
Papule	1 (17)	1 (17)	1 (25)
Pruritus	2 (33)	0	1 (25)
Rash pustular	0	0	1 (25)
Skin burning sensation	1 (17)	0	0
Thermal burn	0	1 (17)	0
Upper respiratory tract infection	1 (17)	0	2 (50)
Viral upper respiratory tract infection	1 (17)	0	0
Vitamin B12 deficiency	0	1 (17)	0
Obese adults			
		Bimagrumab 30 mg/kg (*n* = 6)	Placebo (*n* = 2)
Patients with any AE		6 (100)	1 (50)
Rash		5 (83)	0
Muscle spasms		5 (83)	0
Arthralgia		1 (17)	0
Diarrhoea		1 (17)	0
Dry skin		1 (17)	0
Flank pain		1 (17)	0
Headache		1 (17)	0
Nausea		1 (17)	0
Nephrolithiasis		1 (17)	0
Oral herpes		1 (17)	0
Rhinorrhoea		1 (17)	0
Sinus congestion		2 (33)	0

Adverse events (AEs) ≥1% incidence in any group have been listed; data presented as absolute number of cases (relative percentage). Values are rounded to integer.

Seven of eight (88%) obese participants experienced ≥1 adverse event (*Table* 2), with the most commonly reported being rash and involuntary muscle contractions, referred to as spasms (both 5/8, 63%). Muscle spasms were reported in five of six (83%) participants in the bimagrumab group and none in the placebo group. Adverse events suspected to be related to study drug were reported in 7/8 (88%) participants and included rash and muscle spasms (both 5/8, 63%), oral herpes, dry skin, and diarrhoea (each 1/8, 13%). No serious adverse event was reported in the obese participants.

No bimagrumab‐related, clinically significant electrocardiogram alteration or abnormality was reported in either of the study cohorts. All changes in laboratory test values during the study period were judged to be not clinically significant by the site investigator, and each returned to a normal level prior to study completion.

### Immunogenicity

No anti‐drug antibodies were detected in study participants from either cohort. In older adults, bimagrumab concentrations remained below the lower limit of detection (<0.643 μg/mL) for the end‐of‐study samples in all participants. Conversely, bimagrumab concentrations remained above the lower limit of detection in 5/6 obese participants.

### Pharmacokinetics

**Figure 2:**
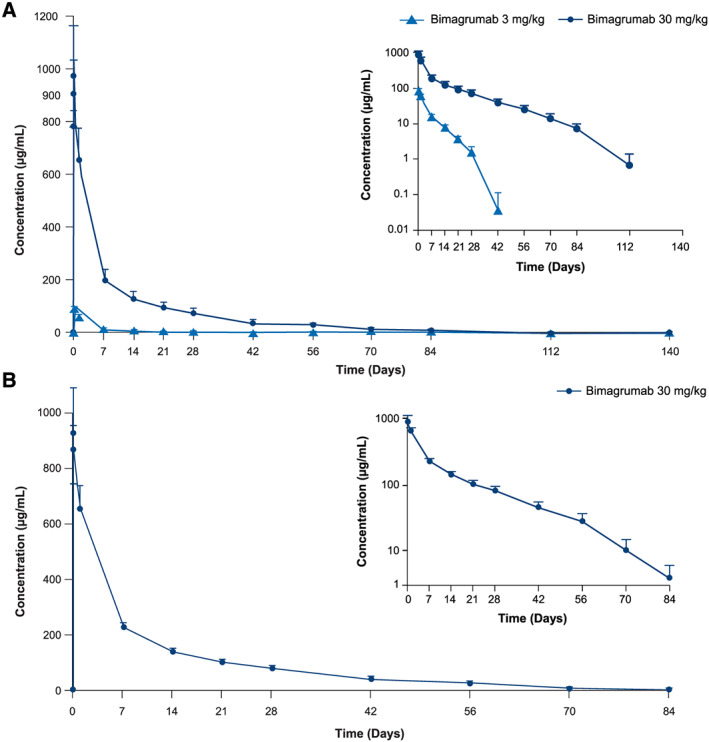
Concentration–time profile of bimagrumab in healthy older adults and obese participants. Data presented as mean (standard deviation). (*A*) Pharmacokinetics in older adults. (*B*) Pharmacokinetics in obese participants. Inset figure shows semi‐logarithmic plot.

The mean plasma concentration–time profile following a single i.v. infusion of bimagrumab 3 or 30 mg/kg is shown in *Figure* 2. Non‐linear PK was observed in concentration profiles of both bimagrumab dose levels. Dose proportionality was observed for C_max_ but not for AUC_last_ (*Table* 3). The PK variability in older adults (~20% CV) and obese participants was relatively low (~10–20% CV) and comparable with that observed in younger healthy volunteers.^15^

**Table 3:** Summary of pharmacokinetic parameters per cohort and treatment

Characteristics		Older adults		Obese adults
		Bimagrumab 3 mg/kg (*n* = 6)	Bimagrumab 30 mg/kg (*n* = 6)	Bimagrumab 30 mg/kg (*n* = 6)
AUC_last_ (μg·day/mL)	Mean ± SD	399 ± 68.7	7320 ± 1490	7650 ± 526
	Range	313–495	5050–8950	6850–8130
C_max_ (μg/mL)	Mean ± SD	90.7 ± 11.3	982 ± 171	944 ± 185
	Range	75.9–105	708–1190	750–1280
T_max_ (h)	Median	4	4	3.1
	Range	2.2–4	2.3–4	2.1–4

AUC_last_, area under the plasma concentration–time curve from time 0 to the time of the last measurable concentration; C_max_, maximum plasma concentration; SD, standard deviation; T_max_, time to reach C_max_.

In older adults administered bimagrumab 3 and 30 mg/kg, mean C_max_ was 90.7 and 982 μg/mL and AUC_last_ was 399 and 7320 μg·day/mL, respectively (*Table* 3). Similarly, obese participants administered bimagrumab 30 mg/kg had a mean C_max_ of 944 μg/mL and AUC_last_ of 7650 μg·day/mL (*Table* 3).

### Pharmacodynamics

**Figure 3:**
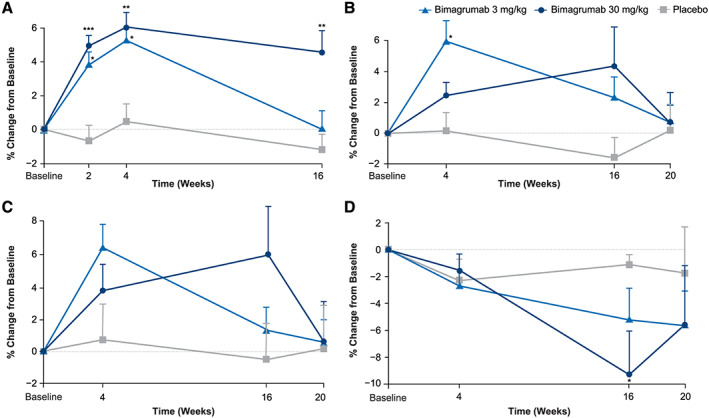
Per cent change from baseline in thigh muscle volume, lean body mass, appendicular lean mass, and fat body mass in older adults. Data presented as mean (standard error). Bimagrumab (3 or 30 mg/kg) vs. placebo, *P < 0.05; **P < 0.01; ^***^ *P* < 0.001. (*A*) Thigh muscle volume. (*B*) Lean body mass. (*C*) Appendicular lean mass. (*D*) Fat body mass.

In healthy older adults, mean (±SD) TMV increased in the bimagrumab 3 mg/kg (3.9 ± 1.8%; 5.3 ± 1.8%) and 30 mg/kg (4.9 ± 1.6%; 6.1 ± 2.2%) groups and remained unchanged in the placebo group (−0.7 ± 1.9%; 0.5 ± 2.1%) at Weeks 2 and 4, respectively (both *P* ≤ 0.02 compared with placebo). At Week 16, TMV values remained elevated in the bimagrumab 30 mg/kg group (4.5 ± 3.3%; *P* ≤ 0.01) and returned to baseline in the bimagrumab 3 mg/kg group (0.01 ± 3.0%) with no change in placebo (−1.2 ± 1.8%) (*Figure* 3). Total LBM did not consistently reflect the increases in TMV. At Week 4, the 3 mg/kg group showed a greater increase in LBM than the 30 mg/kg group (6.0 ± 3.2% vs. 2.4 ± 2.2%; *P* = 0.03, 3 mg/kg compared with placebo), which was driven by a median (range) increase of approximately twice the magnitude [5.9% (1.9 to 10.1%) vs. 3.0% (−1.5 to 4.5%)]. Over the next 12 weeks, LBM decreased in the 3 mg/kg group and continued to increase in the 30 mg/kg group (Week 16: 2.3 ± 3.3% vs. 4.4 ± 6.2%). Comparably, the placebo group showed little change at Weeks 4 and 16 (0.1 ± 2.4% and −1.6 ± 2.6%). At the end of the 12 weeks of follow‐up, LBM returned to near‐baseline values in both bimagrumab groups (*Figure* 3). Throughout the study, changes in appendicular lean mass did not achieve significance in either of the bimagrumab groups compared with placebo. Paralleling LBM changes, participants receiving bimagrumab 3 mg/kg had greater decreases in total FBM at 4 weeks (−2.7 ± 2.9% vs. −1.6 ± 3.0%), but not at 16 weeks (−5.3 ± 5.9% vs. −9.3 ± 8.1%), compared with those receiving bimagrumab 30 mg/kg. The placebo group also showed decreases in total FBM of −2.3 ± 3.2% and −1.1 ± 1.5%. A significant difference in body fat loss was seen between the bimagrumab 30 mg/kg and placebo groups at Week 16 (*P* = 0.02). No change in muscle strength was detected in either bimagrumab group over the study period (*Figure* 3). Changes from baseline in TMV, LBM, FBM, and one‐repetition maximum leg press are shown in *Table* 4.

**Table 4:** Change from baseline in muscle volume, mass, and strength with bimagrumab treatment in older adults

Characteristics/treatment	Bimagrumab 3 mg/kg				Bimagrumab 30 mg/kg				Placebo			
Time (weeks)	0	4	16	20	0	4	16	20	0	4	16	20
TMV (cm^3^)	2406.5 ± 467.7	2533.9 ± 487.7*	2405.8 ± 472.4	NA	2953.2 ± 795.2	3122.8 ± 793.2**	3099.3 ± 893.7**	NA	3225.2 ± 497.0	3242.3 ± 516.2	3184.2 ± 471.3	NA
Total LBM (kg)	29.5 ± 4.5	31.2 ± 4.4*	30.1 ± 3.9	29.6 ± 4.1	36.3 ± 7.6	37.2 ± 7.8	38.1 ± 8.9	36.8 ± 9.1	38 ± 3.1	38.1 ± 3.2	37.4 ± 2.6	38.1 ± 3.5
Total FBM (kg)	27.4 ± 6.6	26.6 ± 6.2	25.9 ± 6.6	25.7 ± 6.4	31.6 ± 9.1	31.2 ± 9.5	29.1 ± 9.8*	30.4 ± 10.6	17.5 ± 3.3	17.1 ± 3.6	17.3 ± 3.5	17.2 ± 3.5
Bilateral leg press (kg)	61.6 ± 13.4	60.1 ± 18.6	73.9 ± 18.6	66.1 ± 18.5	59 ± 13.2	60.2 ± 19.0	60.5 ± 21.0	68.5 ± 16.7	48.8 ± 14.4	57.3 ± 16.0	71.8 ± 13.9	56.7 ± 6.8

Data presented as mean ± standard deviation. Bimagrumab (3 or 30 mg/kg) vs. placebo. TMV and LBM were assessed by analysis of covariance on log‐transformed ratios using the treatment and the log baseline value as covariates. Model was run independently by visit, and values were log back transformed. Analysis of covariance not applied for bilateral leg press. FBM, fat body mass; LBM, lean body mass; NA, not applicable; TMV, thigh muscle volume.

* *P* < 0.05.

** *P* < 0.01.

## Discussion

This study extends the assessment of bimagrumab to two populations of particular interest for activin receptor blockade: the elderly and the obese. Bimagrumab demonstrated a consistent pharmacokinetic profile across the ranges of age and BMI of participants, with periods of concentration‐associated linear and non‐linear clearance suggesting target‐mediated drug disposition. Therefore, other PK parameters, such as clearance and half‐life, could not be derived using non‐compartmental analysis. In older adults, measurable increases in TMV and total LBM, and decreases in total FBM, were seen in both dose levels of bimagrumab, with greater durability of the effects seen with longer exposure time.

Bimagrumab was safe in both older and obese adult men and women. Adverse events centred on tolerability issues involving transient symptoms that spontaneously resolved during the follow‐up period and did not result in an early withdrawal. Two adverse events—submaximal, involuntary skeletal muscle contractions (referred to as muscle spasms) and acne or rash—were observed in both cohorts, with a greater frequency reported by the obese participants. These two symptoms have been reported throughout the development programme of bimagrumab, including in subsequent studies with similar populations.^10^, ^15^, ^16^ It is believed that these muscle symptoms are due to the rapid muscle tissue accretion (measured as TMV and LBM) inducing some intermittent increased myocyte excitability, while the acne or rash may be due to the presence of the ActRII on hair follicles and skin.^17^ Similar muscle spasms have been described with other anabolic treatments, including beta‐agonists, androgens, and resistance training.^18^, ^19^, ^20^

More specifically, studies with other drugs targeting the myostatin–ActRII pathway report overlapping safety profiles. Muscle spasms were reported in the single‐dose study with the anti‐myostatin antibody domagrozumab, in addition to headache, fatigue, and upper respiratory tract infections.^21^ Muscle spasms and rash (both <5% frequency) also were reported in a study of older adult fallers who received 24 weeks of treatment with LY2495655, another anti‐myostatin antibody.^5^ In contrast, the single ascending dose study with ACE‐031, a decoy ActRIIB receptor, observed increased lipase and cholesterol levels, headache, and orthostatic hypotension as the most common adverse events not related to subcutaneous injection but did not report muscle spasms or skin conditions.^4^ While similar muscle‐related adverse events are likely a result of the muscle anabolic effect of the class of drugs, some differences in symptom profiles between bimagrumab and other drugs are likely due to the methods of administration (i.v. infusion vs. subcutaneous injection) and diverse mechanisms of action.

Findings from this study address the question of whether a greater amount of adipose tissue (i.e. higher BMI) would affect the PK of bimagrumab. The ActRII is present on a variety of tissues, including adipocytes.^22^, ^23^ In this study, the pharmacokinetic profiles of the 30 mg/kg dose of bimagrumab were similar in the two study cohorts, suggesting that the dose level provided sufficient receptor binding for physiological efficacy in both populations. While a positive PK–PD relationship was seen in the older adults and not assessed in the obese subjects, a similar PD effect in obese adults has been demonstrated in subsequent studies.^16^, ^24^

The effect of a single dose of bimagrumab on body composition was evaluated in the cohort of older adults. Measurable gains in LBM and TMV were seen with both 3 and 30 mg/kg dose levels resulting in significant increases 4 weeks post dose. Maintenance of the increase in LBM and TMV in the 30 mg/kg group and return to baseline in the 3 mg/kg group at 16 weeks post dose demonstrates the consequence of maintaining or falling below the efficacy threshold of circulating bimagrumab. With target‐mediated drug disposition being the primary mechanism of clearance, the higher dose level (30 mg/kg) was able to maintain a circulating level above the efficacy threshold for a longer period, resulting in a greater durability of the LBM gained.^25^ A corresponding improvement in strength did not follow the increase in LBM in this early study, foreshadowing the majority of studies targeting the myostatin–ActRII pathway that show an uncoupling of an increase in LBM from a gain in strength.^4^, ^5^, ^6^, ^11^, ^26^, ^27^ With preclinical data showing an increase in force generation with hypertrophy,^7^, ^8^ the lack of translation observed may be due to the absence of an effect on mechanical properties of human aging muscle that improve with exercise.^28^ Interestingly, the loss of body fat mass seen with both dose levels of bimagrumab was lowest 16 weeks post dose and remained below baseline values 20 weeks after receiving the single dose.

This study has several limitations. The two cohorts reported here had small sample sizes, there was a gender and body habitus imbalance among the randomized participants, and the study evaluated only a single dose of bimagrumab. While the baseline characteristics are different, no effect on the primary or secondary outcomes was expected. This study was part of the early clinical development programme for bimagrumab and enrolled healthier adults. While both the older adult and obese subjects were well characterized, individuals with severe or uncontrolled chronic or acute disease and women of childbearing potential were excluded for precautionary reasons due to a lack of experience with bimagrumab in humans at the time. Therefore, extrapolation to larger populations of older and obese adults may be limited. However, subsequent studies in various clinical populations (inclusion body myositis, sarcopenia, and type 2 diabetes with obesity) have confirmed the results seen here, although with less intensive PK assessments.^24^, ^26^, ^27^

Bimagrumab was safe and demonstrated consistent PK in healthy older adult and obese men and women. Exposure to a single dose of bimagrumab resulted in a rapid increase in TMV and total LBM and decrease in total body fat, which were sustained in the older adults receiving the highest dose level and longest exposure. The magnitude and duration of muscle hypertrophy and body fat loss were sustained in the older adults to a similar extent to that seen in younger and leaner subjects receiving the same dose level in prior studies. Findings from this study support further examination of bimagrumab in older and obese patient populations with skeletal muscle atrophy and excess adiposity.

## Conflict of interest

D.R., O.P., J.P., M.B., D.L., and R.R. are employees of Novartis and, as such, may be eligible for Novartis stock and stock options. Some of these data were previously presented at 7th International Conference on Frailty & Sarcopenia Research, April 2017, Barcelona, Spain.

## Funding

This study was funded by Novartis Institutes for BioMedical Research, Cambridge, MA, USA, and Basel, Switzerland.

## References

[1] Lee SJ , Reed LA , Davies MV , et al. Regulation of muscle growth by multiple ligands signaling through activin type II receptors. Proc Natl Acad Sci U S A 2005;102:18117–18122. 16330774 10.1073/pnas.0505996102 PMC1306793

[2] Trendelenburg AU , Meyer A , Rohner D , Boyle J , Hatakeyama S , Glass DJ . Myostatin reduces Akt/TORC1/p70S6K signaling, inhibiting myoblast differentiation and myotube size. Am J Physiol Cell Physiol 2009;296:C1258–C1270. 19357233 10.1152/ajpcell.00105.2009

[3] Lee SJ , Lee YS , Zimmers TA , et al. Regulation of muscle mass by follistatin and activins. Mol Endocrinol 2010;24:1998–2008. 20810712 10.1210/me.2010-0127 PMC2954636

[4] Attie KM , Borgstein NG , Yang Y , et al. A single ascending‐dose study of muscle regulator ACE‐031 in healthy volunteers. Muscle Nerve 2013;47:416–423. 23169607 10.1002/mus.23539

[5] Becker C , Lord SR , Studenski SA , et al. Myostatin antibody (LY2495655) in older weak fallers: a proof‐of‐concept, randomised, phase 2 trial. Lancet Diabetes Endocrinol 2015;3:948–957. 26516121 10.1016/S2213-8587(15)00298-3

[6] Rooks DS , Laurent D , Praestgaard J , Rasmussen S , Bartlett M , Tanko LB . Effect of bimagrumab on thigh muscle volume and composition in men with casting‐induced atrophy. J Cachexia Sarcopenia Muscle 2017;8:727–734. 28905498 10.1002/jcsm.12205 PMC5659065

[7] Lach‐Trifilieff E , Minetti GC , Sheppard K , et al. An antibody blocking activin type II receptors induces strong skeletal muscle hypertrophy and protects from atrophy. Mol Cell Biol 2014;34:606–618. 24298022 10.1128/MCB.01307-13 PMC3911487

[8] Morvan F , Rondeau JM , Zou C , et al. Blockade of activin type II receptors with a dual anti‐ActRIIA/IIB antibody is critical to promote maximal skeletal muscle hypertrophy. Proc Natl Acad Sci U S A 2017;114:12448–12453. 29109273 10.1073/pnas.1707925114 PMC5703284

[9] Amato AA , Sivakumar K , Goyal N , et al. Treatment of sporadic inclusion body myositis with bimagrumab. Neurology 2014;83:2239–2246. 25381300 10.1212/WNL.0000000000001070 PMC4277670

[10] Rooks D , Praestgaard J , Hariry S , et al. Treatment of sarcopenia with bimagrumab: results from a phase II, randomized, controlled, proof‐of‐concept study. J Am Geriatr Soc 2017;65:1988–1995. 28653345 10.1111/jgs.14927

[11] Polkey MI , Praestgaard J , Berwick A , et al. Activin type II receptor blockade for treatment of muscle depletion in chronic obstructive pulmonary disease. A randomized trial. Am J Respir Crit Care Med 2019;199:313–320. 30095981 10.1164/rccm.201802-0286OC PMC6363975

[12] Borga M , West J , Bell JD , et al. Advanced body composition assessment: from body mass index to body composition profiling. J Invest Med 2018;66:1–9. 10.1136/jim-2018-000722 PMC5992366 29581385

[13] Borde R , Hortobagyi T , Granacher U . Dose‐response relationships of resistance training in healthy old adults: a systematic review and meta‐analysis. Sports Med 2015;45:1693–1720. 26420238 10.1007/s40279-015-0385-9 PMC4656698

[14] Common Terminology Criteria for Adverse Events (CTCAE) v5.0 USDoHaHS, November 27, 2017 Available at: https://ctep.cancer.gov/protocolDevelopment/electronic_applications/docs/CTCAE_v5_Quick_Reference_8.5x11.pdf. Accessed 16 May 2020.

[15] Garito T , Zakaria M , Papanicolaou DA , et al. Effects of bimagrumab, an activin receptor type II inhibitor, on pituitary neurohormonal axes. Clin Endocrinol (Oxf) 2018;88:908–919. 29566437 10.1111/cen.13601

[16] Garito T , Roubenoff R , Hompesch M , et al. Bimagrumab improves body composition and insulin sensitivity in insulin‐resistant individuals. Diabetes Obes Metab 2018;20:94–102. 28643356 10.1111/dom.13042

[17] Idkowiak‐Baldys J , Santhanam U , Buchanan SM , Pfaff KL , Rubin LL , Lyga J . Growth differentiation factor 11 (GDF11) has pronounced effects on skin biology. PLoS ONE 2019;14:e0218035. 31181098 10.1371/journal.pone.0218035 PMC6557520

[18] Palmer KN . Muscle cramp and oral salbutamol. Br Med J 1978;2:833. 10.1136/bmj.2.6140.833-b PMC1607790 698761

[19] Tomlinson B , Cruickshank JM , Hayes Y , et al. Selective beta‐adrenoceptor partial agonist effects of pindolol and xamoterol on skeletal muscle assessed by plasma creatine kinase changes in healthy subjects. Br J Clin Pharmacol 1990;30:665–672. 1980200 10.1111/j.1365-2125.1990.tb03834.x PMC1368165

[20] Watson SL , Weeks BK , Weis LJ , Harding AT , Horan SA , Beck BR . High‐intensity resistance and impact training improves bone mineral density and physical function in postmenopausal women with osteopenia and osteoporosis: the LIFTMOR randomized controlled trial. J Bone Miner Res 2018;33:211–220. 28975661 10.1002/jbmr.3284

[21] Bhattacharya I , Pawlak S , Marraffino S , et al. Safety, tolerability, pharmacokinetics, and pharmacodynamics of domagrozumab (PF‐06252616), an antimyostatin monoclonal antibody, in healthy subjects. Clin Pharmacol Drug Dev 2018;7:484–497. 28881472 10.1002/cpdd.386

[22] Javelaud D , Mauviel A . Mammalian transforming growth factor‐βs: Smad signaling and physio‐pathological roles. Int J Biochem Cell Biol 2004;36:1161–1165. 15109563 10.1016/S1357-2725(03)00255-3

[23] Rebbapragada A , Benchabane H , Wrana JL , Celeste AJ , Attisano L . Myostatin signals through a transforming growth factor beta‐like signaling pathway to block adipogenesis. Mol Cell Biol 2003;23:7230–7242. 14517293 10.1128/MCB.23.20.7230-7242.2003 PMC230332

[24] Coleman L , Heymsfield S , Miller M , et al. Bimagrumab, an activin receptor antagonist, for treatment of obesity and type 2 diabetes [T‐P‐LB‐3714]. ObesityWeek; November 3–7 2019; Las Vegas, NV.

[25] Keizer RJ , Huitema AD , Schellens JH , Beijnen JH . Clinical pharmacokinetics of therapeutic monoclonal antibodies. Clin Pharmacokinet 2010;49:493–507. 20608753 10.2165/11531280-000000000-00000

[26] Hanna MG , Badrising UA , Benveniste O , et al. Safety and efficacy of intravenous bimagrumab in inclusion body myositis (RESILIENT): a randomised, double‐blind, placebo‐controlled phase 2b trial. Lancet Neurol 2019;18:834–844. 31397289 10.1016/S1474-4422(19)30200-5

[27] Rooks D , Swan T , Goswami B , et al. Safety and efficacy of bimagrumab in community‐dwelling older adults with sarcopenia [O‐66]. 15th Intern Cong European Geria Med Soc; 25–27 September 2019; Krakow, Poland.

[28] Lavin KM , Roberts BM , Fry CS , Moro T , Rasmussen BB , Bamman MM . The importance of resistance exercise training to combat neuromuscular aging. Physiology (Bethesda) 2019;34:112–122. 30724133 10.1152/physiol.00044.2018 PMC6586834

[29] von Haehling S , Morley JE , Coats AJS , Anker SD . Ethical guidelines for publishing in the Journal of Cachexia, Sarcopenia and Muscle: update 2019. J Cachexia Sarcopenia Muscle 2019;10:1143–1145. 31661195 10.1002/jcsm.12501 PMC6818444

